# Effect of a Ketogenic Diet on the Nutritional Parameters of Obese Patients: A Systematic Review and Meta-Analysis

**DOI:** 10.3390/nu13092946

**Published:** 2021-08-25

**Authors:** Miguel Ángel López-Espinoza, Salvador Chacón-Moscoso, Susana Sanduvete-Chaves, María José Ortega-Maureira, Tamara Barrientos-Bravo

**Affiliations:** 1 Facultad de Salud, Universidad Santo Tomás, Talca 3460000, Chile; mlopez34@santotomas.cl (M.Á.L.-E.); mariajoseortegamaureira@gmail.com (M.J.O.-M.); t.barrientos.bravo@gmail.com (T.B.-B.); 2 Experimental Psychology Department, Universidad de Sevilla, 41018 Sevilla, Spain; 3 Departamento de Psicología, Universidad Autónoma de Chile (Chile), Santiago 7500912, Chile

**Keywords:** ketogenic diet, KD, obesity, systematic review, meta-analysis

## Abstract

The effect of a ketogenic diet (KD) on biochemical parameters and nutritional status has been the subject of debate over the years, as several randomized clinical trials (RCTs) obtained different results. Method: A systematic review and random-effects meta-analysis of RCTs comparing KD with a balanced diet was performed by means of a search of PubMed, Cochrane Library, Scopus, and Web of Science. Trials where the method for measuring the response variables was unclear, those that considered pathologies other than chronic non-communicable diseases and those with participants receiving pharmacological treatment for obesity were excluded from the comparison. Results: Of the studies included in the meta-analysis, no statistically significant standardized mean differences were observed for body mass index (BMI) (*d* = −0.457, *p* = 0.359), total cholesterol, COL-T (*d* = 0.230, *p* = 0.591), high-density lipoprotein, HDL (*d* = −0.028, *p* = 0.934), low-density lipoprotein, LDL (*d* = 0.528, *p* = 0.173), or triglycerides, TG (*d* = −0.283, *p* = 0.222), with high values of heterogeneity. The percentage of women included in the studies is a significant moderating variable in terms of BMI ratio (*z* = −6.68, *p* < 0.001) and TG (*z* = −2.27, *p* = 0.023). Conclusion: A KD shows no more benefits on nutritional parameters than a balanced diet, and adverse effects of being on the diet are sometimes reported.

## 1. Introduction

The impact of obesity on people’s well-being and the resulting increase in the risk of chronic non-communicable diseases [1,2,3,4] are well-known facts about the condition. A combination of caloric restriction and physical exercise are the key components to observing a weight loss of approximately 10% in the sixth month of intervention [5].

However, the public often looks to take advantage of diets without understanding their primary objective or potential risks. One popular diet is the ketogenic diet (KD), developed in 1924 by Russell Wilder for the treatment of motor neuron diseases [6,7]; it has also been successfully used for some types of epilepsy [8]. Specifically, a very low carbohydrate ketogenic diet (VLCKD) intervention consists of an increased proportion of fat (44%) and protein (43%) and minimal glycemic intake (<30 g/day, equivalent to approximately 13%) while restricting caloric intake to less than 800 cal/day [9]. The result is a drop in insulin and the onset of a catabolic state of gluconeogenesis and ketogenesis, in which lipids are mobilized from the liver to different tissues (including the brain) and are oxidized, first producing acetoacetate, beta-hydroxybutyrate, and acetone (nutritional ketosis), which are responsible for generating energy through their conversion into acetyl-CoA [10,11]. The circulating ketone bodies rarely exceed the maximum level of 3.0 mmol/L. On other occasions, severe hyperglycemia has been detected, with ketone bodies higher than 20 mmol/L [9]. 

The effects of a KD on cardiovascular variables are controversial [12]. Though there is evidence that a KD has an effect on the orexinergic system, ameliorating body weight and adiposity [13], one meta-analysis [14] found that although a KD fosters a statistically significant decrease in body mass index (BMI), the effect size is clinically marginal. There are also potential risks since fiber is not ingested in the form of whole grains, fruits, and vegetables when a person excludes carbohydrates from their diet, leading to nutritional deficiencies [15] and increasing fasting plasma triglyceride and high-density lipoprotein (HDL) levels, which are associated with increased atherogenesis [16]. This study uses a systematic review and meta-analysis (SR/MA) to evaluate how different types of KD impact the nutritional parameters of obese patients.

## 2. Materials and Method

### 2.1. Design of the Review

This study is a systematic review and meta-analysis (SR/MA) of clinical trials based on the preferred reporting items for systematic reviews and meta-analyses (PRISMA) checklist [17]. Ethical approval was not required for the current study. 

### 2.2. Criteria for Study Inclusion

All randomized controlled trials (RCTs) that used simple or blocked randomization to analyze adult patients over 18 years of age with a clinical diagnosis of obesity (BMI > 30 kg/mt^2^) were included. Open or blind studies that, when possible, applied methods to conceal the randomization sequence were accepted. The intervention took the form of a VLCKD with less than 10% of energy intake from carbohydrates or a low-carbohydrate diet. The comparison group followed a balanced diet to lose weight that could include either a) a conventional very-low energy diet, or VLED (low in total calories), or b) a conventional low-fat diet, or LFD (low fat with less than 30% of energy intake from fats). The search for terms included body composition, nutritional status, waist circumference, lipid profile (total cholesterol, or Col-total; low-density lipoprotein, or LDL; HDL; and Triglycerides, or TG), glucose, and microbiota. The interventions in the comparison groups lasted at least two weeks. Patients undergoing pharmacological treatment for obesity were excluded.

### 2.3. Search Strategy

Between July and September, we used controlled descriptors extracted from the medical subject headings (MeSH) and free-text terms extracted from the subject-specific language. All keywords addressed all of the elements of the PICO (patients, intervention, comparison, and outcome) question format (see Supplementary Material Table S1). 

The following electronic databases were used: PubMed, Cochrane Library, Web of Science, and ClinicalTrials.org (see Supplementary Material Tables S2–S4). We also contacted the authors of the studies included in the SR/MA, as well as other authors of the bibliographic references from the network, in order to request additional materials from the studies that may not have been published. A second motivation was to get in touch with other authors who had published clinical trials that had not been selected for the SR/MA. Likewise, we reviewed the publications available on Google Scholar (more details in Supplementary Material Table S5). Inclusion was not restricted by the age or language of the studies evaluated.

### 2.4. Study Selection

Two previously trained independent researchers (T.B. and M.J.O.) selected articles with information on the subject matter, based on their titles and abstracts, according to the defined eligibility criteria. The articles were then classified into the categories of “included,” “excluded,” and “uncertain.” The methodology section of the articles initially deemed “uncertain” was read in order to then classify these articles as either “included” or “excluded.” The reviewers then compared their classifications; if the two reviewers could not reach a consensus on an evaluated article, they called on a third methodological expert (M.L.E.). At this point, duplicate articles were also discarded. The second stage involved reviewing the full text of the included studies to thoroughly analyze their relevance to the topic under study. This review was also done independently by the two reviewers, who decided whether the articles would ultimately be included in this SR/MA in accordance with the aforementioned eligibility criteria. Any disagreements were once again resolved by consensus, with guidance from a third methodological expert (M.L.E.).

### 2.5. Data Extraction

The same two previously identified researchers independently extracted the study characteristics, population, intervention, and main outcomes from the published articles. The authors of the articles were contacted to obtain additional information when necessary. The CONSORT (consolidated standards of reporting trials) guidelines were applied in order to facilitate critical reading and interpretation of the studies analyzed [17]. The data on TG, Col-total, and the LDL and HDL fractions were presented in mmol/L. Whenever an article reported measures in mg/dL, the amounts were converted [18].

### 2.6. Quality Assessment

The same two investigators then individually assessed the risk of bias according to the domains suggested by the Cochrane Collaboration: random sequence generation, allocation concealment, blinding of participants and personnel, incomplete outcome, and selective outcome reporting [19]. Each investigator recorded their assessment according to one of three alternatives: low, high, or no clear risk of bias. Potential disagreements were analyzed and discussed until a consensus was reached. The Rev. Manager 5.3 program was used for bias risk management [20].

### 2.7. Data Synthesis

For the RCTs that presented clinical homogeneity (participants, intervention, and outcomes), standardized mean differences were calculated in order to obtain average effect sizes with random effect, given that the primary studies reported were a sample and an intra- and inter-study variability was assumed, with a 95% confidence interval. The extent of heterogeneity was measured with *I*^2^ and Cochran’s Q test, with a *p*-value of *p* < 0.10. Prediction intervals were calculated for the mean effect sizes in any population [21]. 

Meta-regressions (mixed-effects) were also obtained in order to explore the potential influence of moderating variables. Comprehensive meta-analysis (CMA) software was used. 

## 3. Results

### 3.1. Search Results

**Figure 1:**
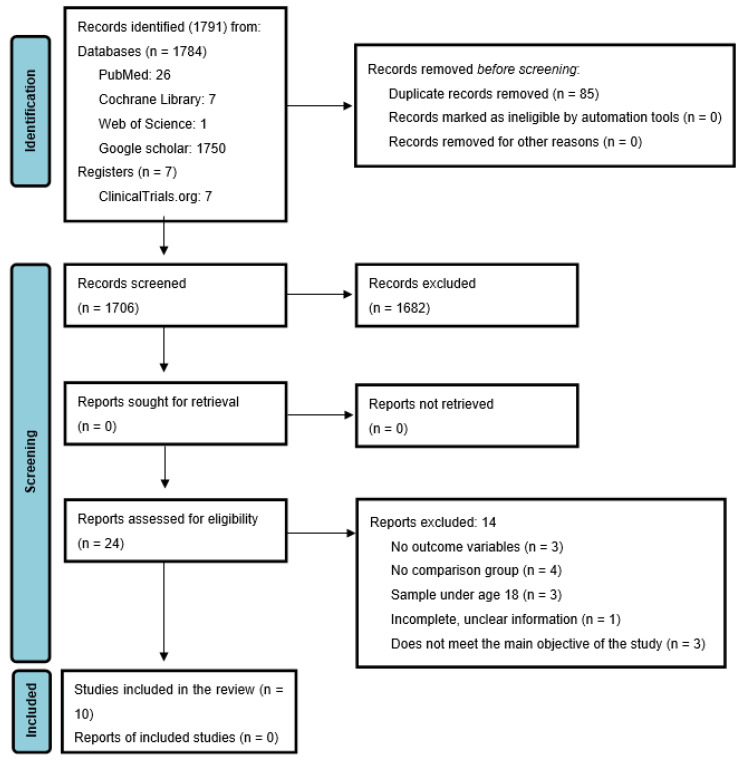
Flow chart of studies included [25].

The search process for eligible articles is presented in Figure 1. Ten studies published in or before 2020 were included and used for description in the qualitative phase, while eight were used to combine their data in the meta-analysis, of which three included data from three different periods [22,23,24]. The primary studies included in the quantitative synthesis are marked with an asterisk in the reference list.

### 3.2. Characteristics of Included Studies

The main characteristics of the studies included are available on Table 1. Three studies were conducted in Spain [22,26,27], three in the United States [23,28,29], two in Australia [30,31], one in Germany [31], and one in Norway [24]. The number of participants assigned to the experimental group ranged from 38 to 270, mean ages ranged from 43 to 60, and the proportion of female participants ranged from 16% to 85%, with the exception of one study that included only men [24]. 

Interventions lasted between four weeks and two years. All studies included interventions with moderate or high-fat and high-protein diets, together with a low percentage of carbohydrates (<50 g CHO/day), with the exception of the study by Haufe 2011 [32], which reported an intake of 90 g/day. The comparison groups ate balanced or modern occidental diets (including highly processed foods). All studies presented two groups. Seven studies [22,24,26,27,30,31,32] assessed BMI, and eight assessed [22,23,24,28,29,30,31,32] HDL, LDL, and TG (Table 2).

### 3.3. Assessment of Risk of Bias

**Figure 2:**
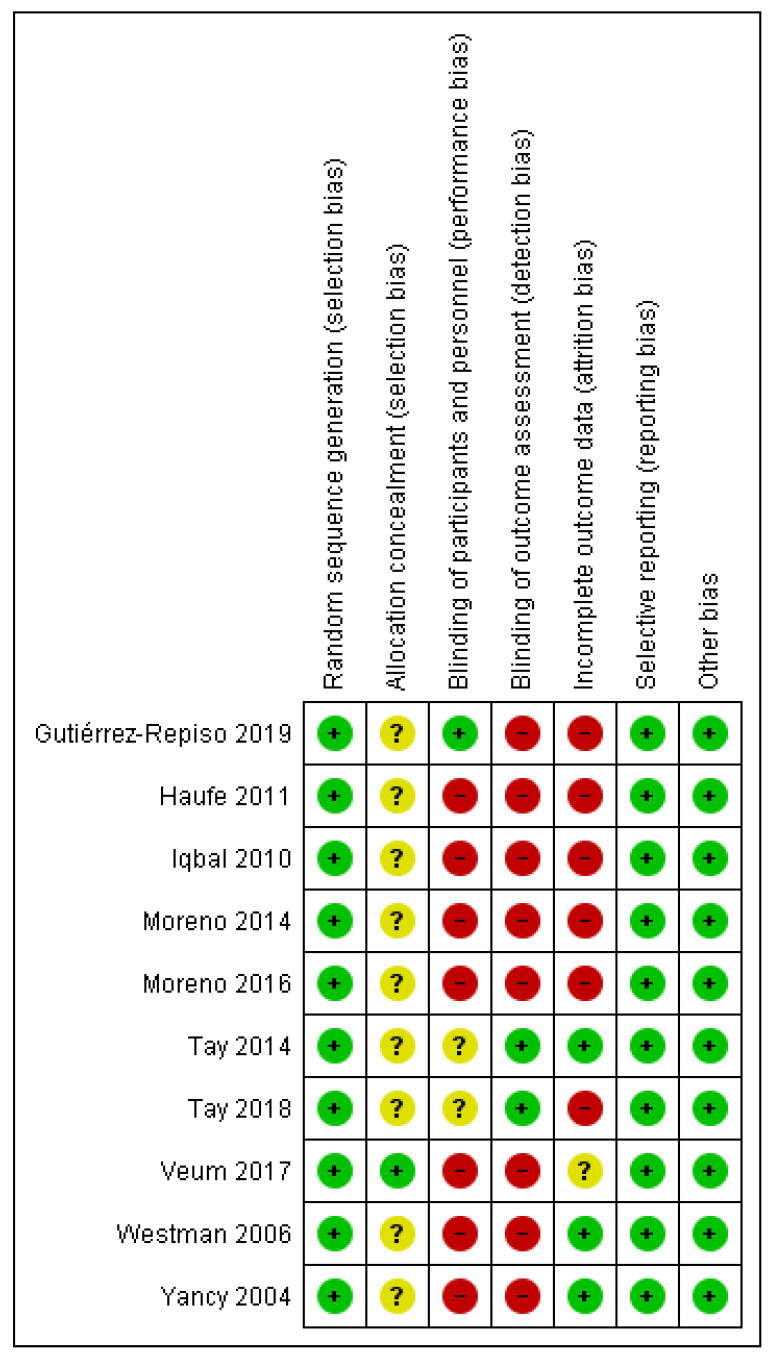
Assessment of the risk of bias of the ten RCT included in this SR. Green: low risk of bias; yellow: unclear risk of bias; and red: high risk of bias.

The assessment of the risk of bias is shown in Figure 2. 

All ten studies included in the SR (see Figure 2) presented low risk of selection bias because they reported details of their random sequence generation. Only Veum 2017 [24] clearly presented the method followed for concealing the allocation sequence; in the rest of the studies, this risk of bias was not clearly controlled. On another note, only Gutiérrez-Repiso 2019 [27] controlled for performance bias; Tay 2014, 2018 [30,31] do not clearly control for this bias, and the rest present the bias given that it was impossible for them to blind patients or evaluators. With regard to the detection bias, the studies by Tay 2014, 2018 [30,31] control for it, while the rest present a high risk of detection bias. With respect to the attrition bias, three presented low risk [28,29,31] while Veum 2017 [24] was unclear; <20% dropout rates were observed, but no reasons were provided. The remaining studies presented a high attrition bias risk [22,23,26,32]. All studies presented a low risk of bias in relation to selective reporting and other biases.

### 3.4. Results of Included Studies

The main results of the studies included are summarized in Table 2. No statistically significant differences in weight loss were found in Tay 2014 [31] at 6 weeks, Iqbal 2010 [23] at 6, 12, and 24 months, Tay 2018 [30] in a 24-month intervention, or Yancy, 2004 [29] at 24 weeks, while Haufe 2011 [32] indicated that there was no statistically significant decrease in BMI. Nevertheless, Moreno 2014 [22] demonstrated weight loss, although the study also described hair loss in the fourth month of intervention. In the first two weeks of the intervention period, the following symptoms were observed: headache, muscle weakness, hyperuricemia (greater than 65 mg/dL), nausea, and leg fatigue. Constipation was observed at two weeks, four months, and one year of intervention. Moreno’s 2016 [26] study reported a 12 kg reduction in body weight after 24 months on a VLCKD versus 4.4 kg in the control group. The Tay 2018 [30] study reported that the estimated glomerular filtration rate remained normal or mildly decreased in both comparison groups.

Yancy 2004 [29], Tay 2014 [31], Westman 2006 [28], and Veum 2017 [24] reported a statistically significant increase in HDL. Yancy 2004 [29] and Tay 2014 [31] reported that LDL levels did not experience a statistically significant decrease.

Westman 2006 [28], Tay 2014 [31], and Yancy 2004 [29] reported statistically significant decreases in TG. Haufe 2011 [32] found no statistically significant differences for HDL, TG, or free fatty acids. LDL and Col-Total did present statistically significant decreases but only in the group that was on the low-fat diet. 

Another finding of interest is that of Gutiérrez-Repiso 2019 [27], who indicated that a VLCKD intervention had no statistically significant effect on intestinal microbiota, which facilitates the production of short-chain fatty acids; however, the diversity of the microbiota did increase significantly (*p* = 0.008). 

### 3.5. Results of the Meta-Analyses

**Figure 3:**
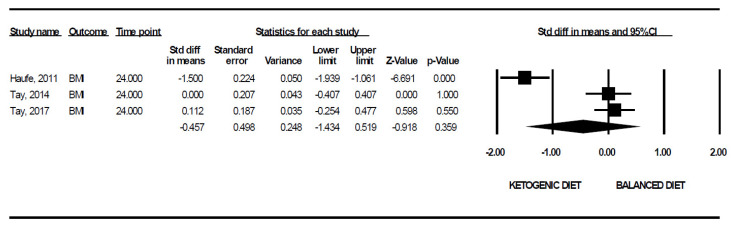
A forest plot of the effect of a ketogenic diet (KD) versus a balanced diet on body mass index (BMI), in kg/mt^2^.

In relation to BMI, only four studies were available. Based on the controlled data of three clinical trials with the same duration of intervention (Figure 3), KD was not found to provoke a significant change in BMI (*d* = −0.46, 95% CI −1.43 to 0.52 kg/mt^2^, *p* = 0.359), with a high heterogeneity value (Q = 35.25, *p* < 0.001, I^2^ = 94.33%). 

**Figure 4:**
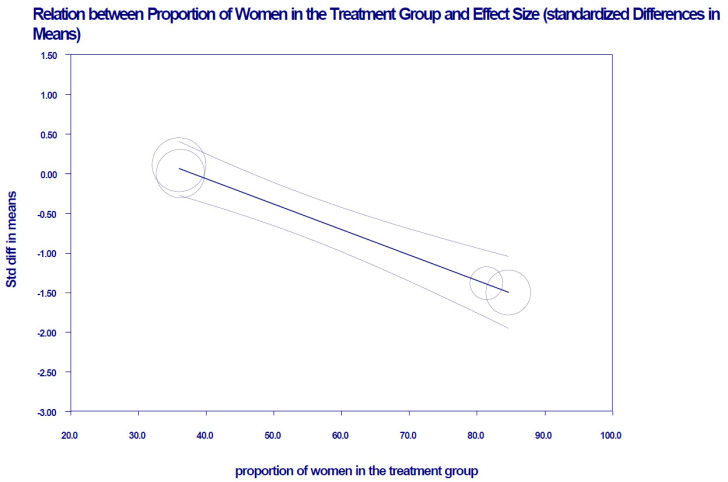
Linear relationship with 95% confidence intervals between the moderating variable “percentage of women” in the meta-analysis studies and the standardized mean observed effect size of BMI (mixed-effects). A decrease in BMI is observed in studies that have included a higher percentage of women.

However, in clinical trials whose samples include a higher percentage of women, the KD intervention decreases BMI, *z* = −6.68, *p* < 0.001 (Figure 4). Supplementary Material: Output S1 shows the meta-regression where this variable was incorporated. 

**Figure 5:**
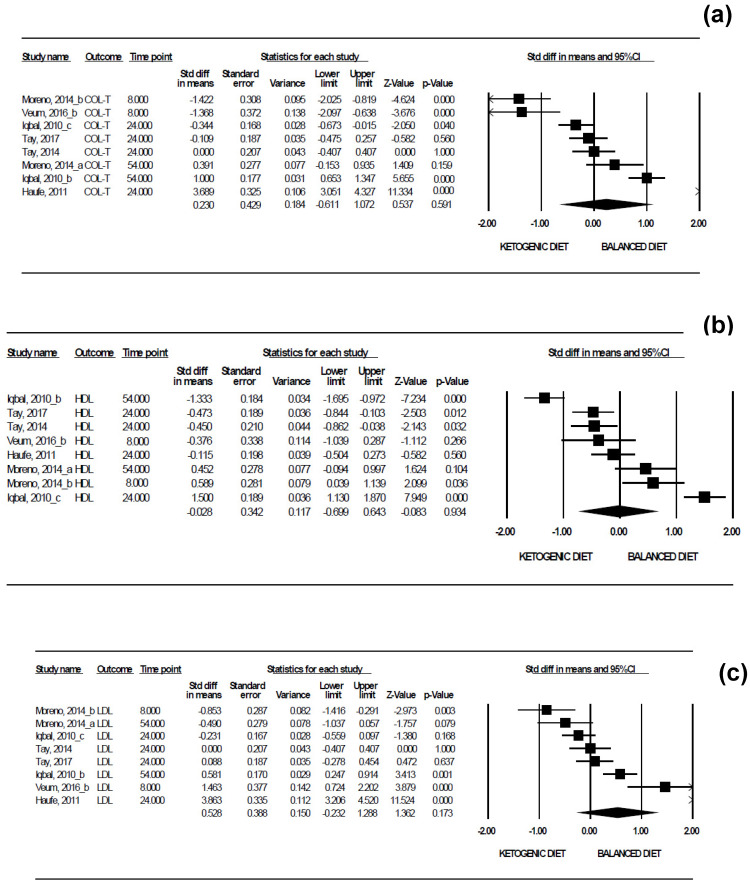

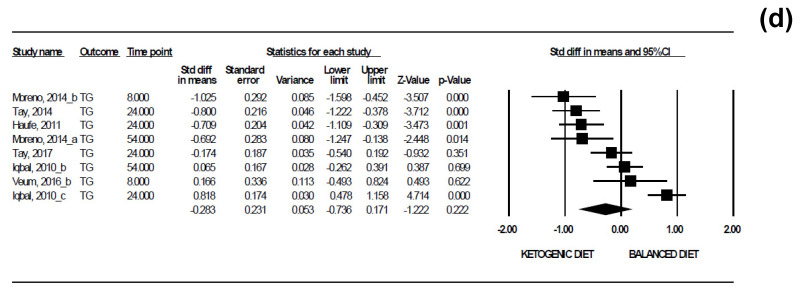
Forest plots showing the meta-analysis of the effects of a KD versus a balanced diet on: (**a**) Total cholesterol (COL-T), (**b**) high-density lipoprotein (HDL), (**c**) low-density lipoprotein (LDL), and (**d**) triglycerides (TG). The individual and mean effect sizes were measured in mmol/L.

**Figure 6:**
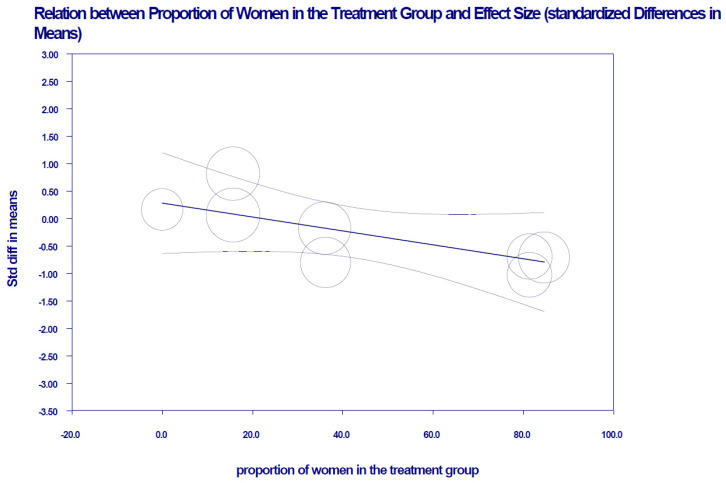
Linear relationship with 95% confidence intervals between the moderating variable “percentage of women” in the meta-analysis studies and the standardized observed mean effect size of TG (mixed-effects). A decrease in TG is observed in studies with a higher percentage of women.

With regard to the effect sizes of a KD on lipid markers where there are at least two studies whose intervention has a similar duration (Figure 5), no statistically significant mean observed effect sizes were found for COL-T (*d* = 0.23, 95% CI −0.61 to 1.07 mmol/L, *p* = 0.591), while the heterogeneity across individual effects was significant (Q = 195.58, *p* < 0.001, I^2^ = 96.42%). The same results were found for HDL (*d* = −0.03, 95% CI −0.70 to 0.64 mmol/L, *p* = 0.934; Q = 13390, *p* < 0.001, I^2^ = 94.77%), LDL (*d* = 0.53, 95% CI −0.23 to 1.29 mmol/L, *p* = 0.173; Q = 162.56, *p* < 0.001, I^2^ = 95.69%), and TG (*d* = −0.28, 95% CI −0.74 to 0.17 mmol/L; *p* = 0.222; Q = 62.87, *p* < 0.001; I^2^ = 88.87%). Adjusting for the duration of intervention, analysis by intention to treat, BMI at baseline, and the proportion of women, no statistically significant mean effects were observed for the first three outcome variables (see Supplementary Material: Output S2, Output S3, and Output S4), with the exception of TG (see Supplementary Material: Output S5), where the variable “proportion of women” presented a statistically significant relationship (z = −2.27, *p* = 0.023), as shown in Figure 6.

No publication bias was detected in any of the analyzed dependent variables: BMI, COL-T, HDL, LDL, and TG (Supplementary Material Figures S1–S5). However, because of the heterogeneity across individual studies, around 60% of the dots are outside the funnel.

Finally, Table 3 presents the prediction intervals for the five response variables. For example, in most of the populations on a KD, the effect on BMI ranges from −14.360 to 13.446, and therefore, is not statistically significant.

## 4. Discussion

The results from the SR/MA aimed to evaluate the effect of different types of KD on nutritional parameters in obese patients over the age of 18. After applying the MA, the results suggest that high-fat diets do not lead to more changes in BMI, COL-T, HDL, LDL, or TG than a balanced diet. Indeed, although BMI presents a combined downward estimate in only three studies, the wide confidence interval range failed to allow for a conclusion of significant effectiveness. This evidence goes against the results published by Bueno et al. [14], who found statistically significant, long-term effects (more than 12 months) for body weight, TG, HDL, and LDL but these were not relevant from a clinical point of view, and even more so if the diet significantly alters eating habits. This evidence contradicts the idea of rapid weight loss [27] in a short period of time, given that a KD is not recommended for more than 12 weeks at a time and that, after completing the diet, the reintroduction of carbohydrates presents a real challenge if weight loss is to be maintained [9]. It, therefore, raises doubts as to whether the ketone bodies are the cause of the weight loss or if instead the decrease in CHO and the KD calorie intake, together with a constant intake of protein that leads to satiety, could be responsible for the temporary weight loss [33,34]. Moreover, a reduction in calories lead to muscle catabolism rather than a loss of adipose tissue [35].

In general, the results of the lipid profile also showed no important clinical effects compared to balanced diets used to reduce obesity. It should be noted that BMI and TG (markers of interest in coronary events and atherosclerosis, respectively) decreased in trials that included a greater proportion of women. In other related studies, there is evidence that the efficacy of KD is tied to sex [36]. Though TG was not expected to decrease in women taking oral contraceptives [37,38], its drop can be attributed to the fact that, in parallel to this group, a decrease in body weight was also observed [39]. This was manifested in BMI, a reduction in appetite and lipogenesis, and an increase in lipolysis, the energy cost of gluconeogenesis, and the thermal effect of proteins [40]. This, in turn, may be explained by the fact that the high-fat foods consumed by the intervention group were high-quality fats (which can be administered as supplements), such as monounsaturated, polyunsaturated [35], and Omega-3 fats, in particular, which decrease blood glucose disorders [41], and thus limit de novo lipogenesis [42]. However, there is no evidence that these changes are long-term, even though there are studies indicating that a KD administered for up to 12 weeks reduces proinflammatory cytokines [9]. 

Although obesity is a risk factor independent from coronary disease, this type of patient presents other comorbidities that require a medically-supervised KD, always supplemented with multivitamins, minerals, and electrolytes. While there may be evidence that a failing heart is related to ketone bodies, the induction of ketone body formation as part of a KD is not conclusive in all studies. Meanwhile, with regard to the effect of ketone bodies on ischemic injuries, evidence only exists at the pre-clinical level [43]. 

Similar to balanced diets consisting mainly of bland fresh foods or meal replacement diet programs, it is important to highlight people’s low tolerance for KD due to the limited palatability of high lipid foods [44]; it is clearly not applicable as a long-term strategy. Moreover, the literature reports increases in uric acid, creatinine, and aspartate aminotransferase [8,45], as well as the short-term symptoms described in the results of this SR. Indeed, if the safety and effectiveness of following a KD cannot be assured [9], it must be recommended with extreme precaution.

Among the limitations to be highlighted in this study is that the MA performed used only pre-post intervention data, and no studies were found to have taken a third post-intervention follow-up measure to study the reversibility of the intervention, as any RCT should demonstrate. In addition, the reported statistical heterogeneity accounts for the different intervention protocols, the characteristics of the subjects, and the intervention durations, which ranged from four weeks to 24 months. This suggests that patients probably completed one or more cycles of KD, interspersing them with other diets. It is also interesting to examine the variability of caloric intake for both interventions and comparators (Table 1). In this meta-analysis, caloric intake could not be considered as a moderator variable because not enough primary studies report on it, nor was there enough data to infer it. Moreover, it is important to highlight that the funnel plots revealed that many of the studies had both high standard error of estimates and high individual effect sizes. This is a possible indicator of the need for large, high-quality trials comparing low-calorie balanced diets with KDs for the treatment of obesity. To the above, we can also add the low number of RCTs retrieved. Notwithstanding, we are confident that as new clinical trials are published, a more robust mean effect size will be assessed.

There are no differential changes between KD and a balanced diet, although it is important to consider that this finding is based on the relatively small number of available studies included in the systematic review and meta-analysis. However, great advances have been made when a KD is prescribed for short periods to treat motor neuron diseases. In contrast, obesity requires a different approach, with a diet that can be maintained over time as a lifestyle, and where a balance is achieved between a progressive improvement of parameters and the avoidance of long-term complications. Furthermore, considering the high cost of diet studies that provide the food for participants, they mostly tend to avoid control groups. Based on these results, and with an eye to future research [46], we suggest a period of intervention that starts before the diet, follows dieters throughout the intervention, and includes a follow up, in order to fully understand the diet’s effects. Moreover, non-adherence, especially when interventions are costly, brings potential side effects that should be considered in meta-analysis [47]. Accordingly, based on the data obtained, it is possible to suggest that balanced dietary interventions can be used to treat obesity effectively, according to WHO recommendations. Based on the available data on body weight reduction and lipid profile improvement, KD is not significantly different from other diets. Furthermore, there could be additional reasons to prescribe a KD to an obese patient beyond those considered in the SM, e.g., the speed of weight loss, patient motivation for further weight loss, the previous failure of balanced diets, or the preparation for surgical interventions.

## Figures and Tables

**Table 1:** Characteristics of studies included.

First Author, Year	Design	Study Population	Intervention	Control	Duration of Intervention	ITT
Moreno, 2016 [26]	An open, prospective, randomized, controlled nutritional intervention clinical trial.	Initial sample Intervention: 22 Control: 23 Mean age ± SD: Intervention: 44.6 ± 7.8 Control: 45.6 ± 9.6 Percentage of women Intervention: 77.3 Control: 95.7 Madrid, Spain	Very low-calorie ketogenic diet (VLCKD): Pronokal method. Three stages. Active stage: 600–800 kcal/day CHO: <50 g/day Protein: 0.80–1.2 g per kilo of ideal weight (only high biological value). In reeducation stage: ketone bodies were evaluated by a physician, and a low-calorie diet was begun, with a progressive incorporation of food. In maintenance: progression to a balanced diet plan.	Low-Calorie Diet (LCD) 10% of the total calories calculated per individual: 1440–1800 kcal/day. Lipids: 25–35% CHO: 45–55% Protein: 15–25% Fiber: 20–40 g/day	24 months	Yes43% total dropout (not specified and cannot be deduced per group)
Moreno, 2014 [22]	An open, prospective, randomized, controlled nutritional intervention clinical trial.	Initial sample Intervention: 27 Control: 26 Mean age ± SD: Intervention: 44.4 ± 8.6 Control: 46.3 ± 9.3 Percentage of women: Intervention: 81.4 Control: 96.1 Madrid, Spain	VLCKD: Pronokal method.	LCD: 10% of the total calories calculated per individual	12 months	Yes Dropout rate Intervention: 30.8% Control: 35.0%
Gutiérrez-Repiso, 2019 [27]	A single-blind, randomized, controlled nutritional intervention clinical trial.	Initial sample Intervention: 15 Control: 9 Mean age ± SD: Intervention: 48.67 ± 9.16 Control: 38.22 ± 11.27 Percentage of women: Intervention: 81.4 Control: 66.6 Málaga, Spain	VLCKD: Pronokal method, plus symbiotic supplements in the form of capsules (2 months) + LCD for 2 months + symbiotics.	Placebo	4 months	Unclear Dropout rate Not reported and cannot be deduced
Westman, 2006 [28]	Two-armed randomized trial	Sample: Intervention: 59 Control: 60 Mean age ± SD: Intervention: 44.4 ± 10.1 Control: 45.6 ± 9.0 Percentage of women: Intervention: 75% Control: 78% North Carolina, USA	Low-carbohydrate, ketogenic diet (initially <20 g of carbohydrates/day) plus nutritional supplements	Low-fat, low-calorie diet	6 months	Unclear Dropout rate: 0.84% total dropout (not specified and cannot be deduced per group) However, the authors consider the initial and final sample to be the same number of participants.
Tay, 2018 [30]	Two-armed randomized trial.	Initial sample Intervention: 58 Control: 57 Mean age ± SD: Intervention: 58 Control: 58 Percentage of women: Intervention: 64% Control: 54% Adelaide, Australia	Low-carbohydrate diet, high in unsaturated fats and low in saturated fats: CHO: 14% (<50 g/day) Protein: 28% Lipids: 58% (35% monounsaturated and 13% polyunsaturated fats)	CHO: 53% (processed foods are discouraged). Protein: 17% Lipids: 30% (15% monounsaturated and 9% polyunsaturated fats)	24 months	Yes Dropout rate: Intervention: 13.8% Control: 15.8%
Veum, 2017 [24]	Two-armed randomized trial.	Initial sample Intervention: 18 Control: 20 Mean age ± SD: Intervention: 40.2 ± 4.5 Control: 40.3 ± 5.5 The study only considered men. Bergen, Norway	High-fat, low-carbohydrate diet: Lipids: 73% CHO: 10%	Low-fat, high-carbohydrate diet: Lipids: 30% CHO: 53%	12 weeks (3 months)	Yes (per protocol principle was also reported) Dropout rate: 17.4% total dropout (not specified and cannot be deduced per group)
Tay, 2014 [31]	Two-armed parallel randomized trial.	Initial sample Intervention: 58 Control: 57 Mean age ± SD: Intervention: 58 ± 7 Control: 58 ± 7 Percentage of women: Intervention: 36.2% Control: 49.1% Adelaide, Australia	Low-carbohydrate diet, high in unsaturated fats and low in saturated fats: CHO: 14% (<50 g/day) Protein: 28% Lipids: 58% (35% monounsaturated and 13% polyunsaturated fats)	CHO: 53% (processed foods are discouraged). Protein: 17% Lipids: 30% (15% monounsaturated and 9% polyunsaturated fats) Saturated fatty acids were restricted to 10% in both groups.	24 weeks (6 months)	Unclear Dropout rate: Intervention: 21% Control: 18%
Haufe, 2011 [32]	Two-armed randomized trial.	Initial sample Intervention: 84 Control: 86 Mean age: Intervention: 43.2 Control: 45.1 Percentage of women: Intervention: 84.6% Control: 80.0% Germany	1200 cal/day diet CHO: 90 g/day Protein: 0.80 g-Kg/day Lipids: >30%	Control diet: Protein: 0.80 g-Kg/day Lipids: 20%	6 months	Yes Dropout rate: Intervention: 38.1% Control: 41.8%
Iqbal, 2010 [23]	Randomized controlled trial	Initial sample Intervention: 70 Control: 74 Mean age: Intervention: 60.0 ± 8.9 Control: 60.0 ± 9.5 Percentage of women: Intervention: 15.7% Control: 5.4% Philadelphia, USA	Low-carbohydrate (high-fat) diet: CHO: <30 g/day Subjects were advised to consume whole and high fiber content foods. Fat intake was not restricted (subjects were advised to consume healthy sources of fat).	Low-fat control diet, <30% of calories/day. Subjects were encouraged to consume healthy fats: <7% of total calories from saturated fat, <300 mg of cholesterol, and to increase intake of fruits and vegetables.	24 months	Yes Dropout rate: Intervention: 60.0% Control: 46.0%
Yancy, 2004 [29]	Two-armed randomized trial	Initial sample Intervention: 60 Control: 60 Mean age: Intervention: 45.6 ± 9.0 Control: 44.2 ± 10.0 Percentage of women: Intervention: 78% Control: 75% North Carolina, USA	Low-carbohydrate diet CHO: <20 g/day (in the beginning) + nutritional supplements + exercise recommendation + group meetings (4 times/month in the beginning and then monthly for three months)	Low-fat diet: Lipids: <30% of energy from fat, <300 mg cholesterol/day, and deficit of 500 to 1000 cal/day + recommendation of exercise + group meetings (4 times/month in the beginning and then monthly for three months).	24 weeks	Unclear Dropout rate: Intervention: 24.0% Control: 43.0%

Abbreviations: ITT, intention to treat; CHO, carbohydrates; VLCKD, Very low-calorie ketogenic diet; LCD, Low-Calorie Diet.

**Table 2:** Results of the studies included.

First Author, Year	BMI	COL-T	HDL	LDL	TG	Side Effects
Gutiérrez-Repiso, 2019 [27]	Proteobacteria and BMI reduction: β = 0.362; *p* < 0.038	---	--	--	--	Not reported
Haufe, 2011 [32]	I(post-pre): −2.7 ± 0.2	I(post-pre): −0.08 ± 0.09	I(post-pre): −0.09 ± 0.1	I(post-pre): −0.04 ± 0.07	I(post-pre): −0.19 ± 0.06	Not reported
	C(post-pre): −2.4 ± 0.2	C(post-pre): −0.45 ± 0.11	C(post-pre): −0.1 ± 0.07	C(post-pre): −0.33 ± 0.08	C(post-pre): −0.14 ± 0.08	
Westman, 2006 [28]	--	I(post-pre): −0.21	I(post-pre): 0.14	I(post-pre): 0.04	I(post-pre): −0.84	Not reported
		C(post-pre): −0.35	C(post-pre): −0.04	C(post-pre): −0.19	C(post-pre): −0.32	
Moreno, 2014 [22]	54 weeks I(post-pre): −7.0 ± 3.9 C(post-pre): −2.6 ± 2.2	54 weeks	54 weeks	54 weeks	54 weeks	Statistically significant symptoms: After 2 weeks: asthenia, headache, muscle weakness, constipation, hyperuricemia, nausea, heaviness, and fatigue in the legs. At 4 months: Hair loss, constipation. At 12 months: Constipation.
		I1: 5.36 ± 0.99	I1: 1.57 ± 0.47	I1: 3.08 ± 0.93	I1: 1.56 ± 1.01	
		I2: 4.99 ± 1.18	I2: 1.77 ± 0.39	I2: 2.72 ± 0.87	I2: 1.01 ± 1.13	
		C1: 4.81 ± 0.99	C1: 1.38 ± 0.33	C1: 2.92 ± 0.75	C1: 1.09 ± 0.40	
		C2: 4.76 ± 1.01	C2: 1.44 ± 0.37	C2: 2.87 ± 0.67	C2: 0.99 ± 0.51	
			
		8 weeks	8 weeks	8 weeks	8 weeks	
		I1: 5.36 ± 0.99	I1: 1.57 ± 0.47	I1: 3.08 ± 0.93	I1: 1.56 ± 1.01	
		I2: 4.1 ± 0.76	I2: 1.24 ± 0.27	I2: 2.41 ± 0.62	I2: 1.01 ± 0.40	
		C1: 4.81 ± 0.99	C1: 1.38 ± 0.33	C1: 2.92 ± 0.75	C1: 1.09 ± 0.40	
		C2: 4.56 ± 0.76	C2: 1.23 ± 0.35	C2: 2.80 ± 0.78	C2: 1.16 ± 0.44	
			
		16 weeks	16 weeks	16 weeks	16 weeks	
		I1: 5.36 ± 0.99	I1: 1.57 ± 0.47	I1: 3.08 ± 0.93	I1: 1.56 ± 1.01	
		I2: 4.55 ± 0.77	I2: 1.39 ± 0.33	I2: 2.77 ± 0.63	I2: 0.87 ± 0.26	
		C1: 4.81 ± 0.99	C1: 1.38 ± 0.33	C1: 2.92 ± 0.75	C1: 1.09 ± 0.40	
		C2: 4.63 ± 1.00	C2: 1.28 ± 0.35	C2: 2.84 ± 0.87	C2: 1.14 ± 0.47	
Moreno, 2016 [26]	I: −4.4 C: −1.9	--	--	--	--	Asthenia, fatigue, headaches, constipation, and nausea.
Iqbal, 2010 [23]	--	96 weeks	96 weeks	96 weeks	96 weeks	Two deaths in the intervention group and three deaths in the control group.
		I(post-pre): −0.31 ± 0.18	I(post-pre): 0.02 ± 0.03	I(post-pre): −0.2 1 ± 0.16	I(post-pre): −0.29 ± 0.14	
		C(post-pre):	C(post-pre):	C(post-pre):	C(post-pre):	
		−0.34 ± 0.16	0.02 ± 0.03	−0.16 ± 0.14	−0.15 ± 0.14	
			
		54 weeks	54 weeks	54 weeks	54 weeks	
		I(post-pre): −0.02 ± 0.19	I(post-pre): 0.07 ± 0.03	I(post-pre): −0.12 ± 0.16	I(post-pre): −0.14 ± 0.16	
		C(post-pre):	C(post-pre):	C(post-pre):	C(post-pre):	
		−0. 21 ± 0.19	0.03 ± 0.03	−0.21 ± 0.15	−0.15 ± 0.15	
			
		24 weeks	24 weeks	24 weeks	24 weeks	
		I(post-pre): 0.03 ± 0.14	I(post-pre): 0.01 ± 0.04	I(post-pre): 0.02 ± 0.13	I(post-pre): −0.01 ± 0.11	
		C(post-pre):	C(post-pre):	C(post-pre):	C(post-pre):	
		−0.02 ± 0.15	0.07 ± 0.04	−0.05 ± 0.13	−0.1 ± 0.11	
Tay, 2014 [31]	I(post-pre): −4.0 ± 2.0	I(post-pre): −0.3 ± 0.7	I(post-pre): 0.03 ± 0.2	I(post-pre): −0.3 ± 0.5	I(post-pre): −0.5 ± 0.5	Intervention group: Constipation and diverticulitis; prostate cancer.
	C(post-pre): −4.0 ± 1.8	C(post-pre): −0.3 ± 0.9	C(post-pre): −0.06 ± 0.2	C(post-pre): −0.3 ± 0.7	C(post-pre): −0.1 ± 0.5	Control group: Esophageal ulcer.
Tay, 2018 [30]	I(post-pre):	I(post-pre):	I(post-pre):	I(post-pre):	I(post-pre):	Not reported.
	−2.1 (−2.8; −1.5) **	0.2 (−0.1; 0.6) **	0.02 (−0.05; 0.1)	0.2 (−0.1; 0.5)	−0.1 (−0.3; 0.2)	
	C(post-pre):	C(post-pre):	C(post-pre):	C(post-pre):	C(post-pre):	
	−2.3 (−3.0; −1.6) **	0.1 (−0.3 to 0.4) **	−0.1 (−0.1; 0.01) **	0.1 (−0.2; 0.4) **	0.1 (−0.2; 0.3) **	
Veum, 2017 [24]	I(post-pre)–C(post-pre): −3.6 (−4.04; −3.18) **	I(post-pre):	I(post-pre):	I(post-pre):	I(post-pre):	Not reported.
		−0.13 (−0.29; 0.55)	0.14 (0.06; 0.22)	0.26 (−0.08; 0.60)	−0.53 (−0.68; −0.37)	
		C(post-pre):	C(post-pre):	C(post-pre):	C(post-pre):	
		−0.96 (−1.23; −0.69) **	−0.01 (−0.10; 0.07) **	−0.78 (−1.08; −0.49) **	−0.41 (−0.60; −0.21) **	
			
		8 weeks	8 weeks	8 weeks	8 weeks	
		I1: 5.35 ± 1.17	I1: 1.05 ± 0.3	I1: 3.65 ± 1.14	I1: 1.52 ± 0.6	
		I2: 5.78 ± 1.22	I2: 1.13 ± 0.27	I2: 4.19 ± 1.18	I2: 1.26 ± 0.57	
		C1: 5.42 ± 1.14	C1: 1.23 ± 0.24	C1: 3.68 ± 1.07	C1: 1.45 ± 0.53	
		C2: 4.64 ± 0.95	C2: 1.23 ± 0.27	C2: 2.98 ± 0.89	C2: 1.12 ± 0.37	
			
		4 weeks	4 weeks	4 weeks	4 weeks	
		I1: 5.35 ± 1.17	I1: 1.05 ± 0.3	I1: 3.65 ± 1.14	I1: 1.52 ± 0.6	
		I2: 5.56 ± 1.23	I2: 1.1 ± 0.21	I2: 3.99 ± 1.15	I2: 1.22 ± 0.48	
		C1: 5.42 ± 1.14	C1: 1.23 ± 0.00.24	C1: 3.68 ± 1.07	C1: 1.45 ± 0.53	
		C2: 4.6 ± 0.94	C2: 1.23 ± 0.29	C2: 2.99 ± 0.86	C2: 1.18 ± 0.54	
Yancy, 2004 [29]	--	I(post-pre):	I(post-pre):	I(post-pre):	I(post-pre):	Intervention group:
		−0.21	0.14	0.04	−0.84	Constipation, headache, halitosis, muscle cramps, diarrhea, general weakness, and skin rash.
		C(post-pre):	C(post-pre):	C(post-pre):	C(post-pre):	Control group:
		−0.35	−0.04	−0.19	−0.31	One patient developed heart disease.

Abbreviations: COL-T, total cholesterol; HDL, high-density lipoprotein; LDL, low-density lipoprotein; TG, triglycerides; BMI, body mass index; I, intervention group; C, control group. 1, pre; 2, post; ** 95% confidence interval. All values are indicated in mmol/L.

**Table 3:** Prediction intervals for estimating the actual mean difference in MA.

Outcome	Number of Studies	Tau ^2^	Prediction Intervals
BMI	3	0.9487	−14.360; 13.446
Col-T	8	1.4040	−2.854; 3.314
HDL	8	0.8756	−2.466; 2.401
LDL	8	1.1399	−2.251; 3.307
TG	8	0.3735	−1.882; 1.316

Calculation takes into account the number of studies, tau ^2^, mean difference, and the upper confidence limit reported by the MA. Abbreviations: BMI, body mass index; COL-T, total cholesterol; HDL, high-density lipoprotein; LDL, low-density lipoprotein; and TG, triglycerides.

## Data Availability

The data presented in this study are available on request from the corresponding author.

## Supplementary Materials

The following are available online at https://www.mdpi.com/article/10.3390/nu13092946/s1, Table S1: Keywords and their relationship to PICO, Table S2: PubMed search strategy, Table S3: Cochrane Library search strategy, Table S4: Web of Science search strategy, Table S5: ClinicalTrials.org search strategy, Table S6: Google Scholar search strategy, Output S1: Meta-regression between KD and BMI with the moderator “proportion of women in the study”, Output S2: Meta-regression between KD and COL-T with moderators “proportion of women in the study”, “BMI at baseline”, “intention-to-treat analysis” and “weeks of intervention”, Output S3: Meta-regression between KD and HDL with moderators “proportion of women in the study”, “BMI at baseline”, “intention-to-treat analysis” and “weeks of intervention”, Output S4: Meta-regression between KD and LDL with moderators “proportion of women in the study”, “BMI at baseline”, “intention-to-treat analysis” and “weeks of intervention”, Output S5: Meta-regression between KD and TG with moderators “proportion of women in the study”, “BMI at baseline”, “intention-to-treat analysis” and “weeks of intervention”; Figure S1: Funnel Plot-Dependent Variable BMI; Figure S2: Funnel Plot-Dependent Variable COL-T; Figure S3: Funnel Plot-Dependent Variable HDL; Figure S4: Funnel Plot-Dependent Variable LDL; Figure S5: Funnel Plot-Dependent Variable TG.
