# Supplementary material for: Effect of a Ketogenic Diet on the Nutritional Parameters of Obese Patients: A Systematic Review and Meta-Analysis
Source: Nutrients. 2021 Aug 25;13(9):2946. doi: 10.3390/nu13092946 (PMC8467306; doi:10.3390/nu13092946)

Supplementary Material

Table S1. Keywords and their relationship to PICO

Strategy	Free keywords	MeSH
Patient	Adult*	“Adult”<br>“Obesity”
Intervention	Ketogenic Diet	“Diet, ketogenic”
Control	“Low-Calorie Diet”	“Caloric Restriction"<br>“Diet, Fat-Restricted"
Outcome	Quetelet Index<br>“LDL Lipoproteins”<br>“Low-Density Lipoproteins”<br>“LDL-1”<br>High Density Lipoproteins<br>Lipoproteins<br>Triacylglycerol*<br>Microbiome<br>“Human Microbiome”	“Body Mass Index”<br>"Body Composition"<br>“Cholesterol”<br>"Lipoproteins, LDL"<br>“Lipoproteins, HDL"<br>“Triglycerides”<br>“Blood Glucose”<br>“Microbiota”[MeSH]<br>“Appetite”[MeSH]<br>“Satiation”[MeSH]

Table S2. PubMed search strategy: [26]

Search number	Query	Results
#27	Search: #23 AND #24 AND #25 AND #26 AND (clinicaltrial[Filter]) Filters: Clinical Trial Sort by: Most Recent	26
#26	Search: #9 OR #10 OR #11 OR #12 OR #13 OR #14 OR #15 OR #16 OR #17 OR #18 OR #19 OR #20 OR #21 OR #22 Sort by: Most Recent	737,419
#25	Search: #6 OR #7 OR #8 Sort by: Most Recent	41,956
#24	Search: #4 OR #5 Sort by: Most Recent	3,473
#23	Search: #1 OR #2 AND #3 Sort by: Most Recent	111,901
#22	Search: satiation[MeSH Terms] Sort by: Most Recent	5,783
#21	Search: appetite[MeSH Terms] Sort by: Most Recent	10,650
#20	Search: Human Microbiome Sort by: Most Recent	76,590
#19	Search: Microbiome Sort by: Most Recent	87,082
#18	Search: microbiota[MeSH Terms] Sort by: Most Recent	40,938
#17	Search: blood glucose[MeSH Terms] Sort by: Most Recent	167,013
#16	Search: Triacylglycerol* Sort by: Most Recent	16,989
#15	Search: triglycerides[MeSH Terms] Sort by: Most Recent	77,849
#14	Search: lipoproteins, hdl[MeSH Terms] Sort by: Most Recent	44,283
#13	Search: lipoproteins, ldl[MeSH Terms] Sort by: Most Recent	54,647
#12	Search: Cholesterol[MeSH Terms] Sort by: Most Recent	161,947
#11	Search: body composition[MeSH Terms] Sort by: Most Recent	56,446
#10	Search: Quetelet Index Sort by: Most Recent	256,934
#9	Search: Body Mass Index[MeSH Terms] Sort by: Most Recent	130,782
#8	Search: Diet, Fat-Restricted[MeSH Terms] Sort by: Most Recent	3,732
#7	Search: Caloric Restriction[MeSH Terms] Sort by: Most Recent	6,263
#6	Search: Low-Calorie Diet Sort by: Most Recent	38,900
#5	Search: Ketogenic Diet Sort by: Most Recent	3,473
#4	Search: diet, ketogenic[MeSH Terms] Sort by: Most Recent	1,440
#3	Search: Obesity[MeSH Terms] Sort by: Most Recent	219,668
#2	Search: Adult[MeSH Terms] Sort by: Most Recent	7,365,197
#1	Search: Adult* Sort by: Most Recent	6,024,174

Table S3. Cochrane Library search strategy: [7]

ID	Search	Hits
#1	MeSH descriptor: [Obesity] explode all trees	13727
#2	adult*	655526
#3	#1 AND #2	8931
#4	MeSH descriptor: [Diet, Ketogenic] explode all trees	58
#5	Ketogenic	488
#6	#4 OR #5	488
#7	MeSH descriptor: [Caloric Restriction] explode all trees	769
#8	MeSH descriptor: [Diet, Fat-Restricted] explode all trees	962
#9	#7 OR #8	1685
#10	MeSH descriptor: [Body Mass Index] explode all trees	10061
#11	cholesterol	36822
#12	MeSH descriptor: [Lipoproteins] explode all trees	9690
#13	LDL	20821
#14	HDL	16861
#15	MeSH descriptor: [Triglycerides] explode all trees	6301

#16	MeSH descriptor: [Blood Glucose] explode all trees	15950
#17	MeSH descriptor: [Microbiota] explode all trees	676
#18	Human Microbiome	1354
#19	MeSH descriptor: [Appetite] explode all trees	1441
#20	Appetite Alterations	191
#21	MeSH descriptor: [Satiation] explode all trees	1043
#22	#10 OR #11 OR #12 OR #13 OR #14 OR #15 OR #16	
OR #17 OR #18 OR #19 OR #20 OR #21		65625
#23	#3 AND #6 AND #9 AND #22	7

Table S4. Web of Science search strategy: [1]

5 1

#4 AND #3 AND #2 AND #1

Índices=SCI-EXPANDED, SSCI, A&HCI, ESCI Período de tiempo=Todos los años

4 822.833

TS=(Body Mass Index OR Cholesterol OR Lipoproteins OR LDL OR HDL OR Triglycerides OR Blood Glucose OR Microbiota OR Human Microbiome OR Appetite OR Appetite Alterations OR Satiation)

Índices=SCI-EXPANDED, SSCI, A&HCI, ESCI Período de tiempo=Todos los años

3 9.774

TS=(Caloric, Restriction OR Diet, Fat-Restricted)

Índices=SCI-EXPANDED, SSCI, A&HCI, ESCI Período de tiempo=Todos los años

2 4.808

TS= (Ketogenic OR Diet, Ketogenic)

Índices=SCI-EXPANDED, SSCI, A&HCI, ESCI Período de tiempo=Todos los años

1 62.618

TS=(adult* AND obesity)

Índices=SCI-EXPANDED, SSCI, A&HCI, ESCI Período de tiempo=Todos los años

Table S5. ClinicalTrials.org search strategy: [7]

Biochemical parameters | Ketogenic Dieting	0
Nutritional status | Ketogenic Dieting	7

Table S6. Google Scholar search strategy: [1750]

(adult*) AND ("obesity") AND ("diet, ketogenic" OR "ketogenic diet" OR "diets, ketogenic") AND ("caloric restriction") AND ("body mass index" OR "cholesterol" OR “lipoproteins” OR “HDL” OR “LDL” OR “triglycerides” OR “microbioma”) AND (“Randomized Controlled Trial”)

Output S1.

Meta-regression between VLCKD and BMI with the Moderator "Proportion of Women in the Study"

Main results for Model 1, Random effects (MM), Z-Distribution, Std diff in means

Covariate	Coefficient	Standard Error	95% Lower	95% Upper	Z-value	2-sided P-value
Intercept	1.2203	0.2803	0.6709	1.7697	4.35	0
women prop	-0.0321	0.0048	-0.0415	-0.0227	-6.68	0

Statistics for Model 1

Test of the model: Simultaneous test that all coefficients (excluding intercept) are zero

Q = 44.63, df = 1.0 p = 0.0000
Goodness of fit: Test that unexplained variance is zero

Tau^2 = 0.0000, Tau = 0.0000, I^2 = 0.00%, Q = 0.14, df = 2.0 p = 0.9312

Comparison of Model 1 with the null model

Total between-study variance (intercept only)

Tau^2 = 0.6982, Tau = 0.8356, I^2 = 93.30%, Q = 44.77, df = 3.0 p = 0.0000

Proportion of total between-study variance explained by Model 1

R^2 analog = 1.00

Output S2.

Meta-Regression between VLCKD and Col-Total with Moderators “Proportion of Women in the Study," “BMI at Baseline," “Intention-to-Treat Analysis,” and “Weeks of Intervention"

Main results for Model 1, Random effects (MM), Z-Distribution, Std diff in means

Covariate	Coefficient	Standard	95%	95%	Z-value	2-sided
		Error	Lower	Upper		P-value
		Error	Lower	Upper	P-value	
Intercept	10.7755	18.3428		-25.1756		46.7267
	0.59 0.5569					
women prop	0.0103	0.0207	-0.0303	0.0509	0.5	0.6198
initial BMI	-0.3555	0.5318	-1.3978	0.6868	-0.67	0.5038
Itt: yes	0.2336	1.7459	-3.1883	3.6556	0.13	0.8935
Intercept	0.048	0.0406	-0.0317	0.1277	1.18	0.2375

Statistics for Model 1

Test of the model: Simultaneous test that all coefficients (excluding intercept) are zero

Q = 2.52, df = 4.0 p = 0.6415
Goodness of fit: Test that unexplained variance is zero
Tau^2 = 2.4561, Tau = 1.5672, I^2 = 97.65%, Q = 127.60, df = 3.0 p = 0.0000

Comparison of Model 1 with the null model

Total between-study variance (intercept only)
Tau^2 = 1.4059, Tau = 1.1857, I^2 = 96.42%, Q = 195.58, df = 7.0 p = 0.0000

Proportion of total between-study variance explained by Model 1

R^2 analog = 0.00 (computed value is -0.75)

Output S3.

Meta-Regression between VLCKD and HDL with Moderators “Proportion of Women in the Study," “BMI at Baseline," “Intention-to-Treat Analysis,” and “Weeks of Intervention"

Main results for Model 1, Random effects (MM), Z-Distribution, Std diff in means

Covariate	Coefficient	Standard	95%	95%	Z-value	2-sided
		Error	Lower	Upper		P-value
		Error	Lower	Upper	P-value	
Intercept	-13.9527	11.2564		-36.0148		8.1093
-1.24	0.2151					
women prop	0.0146	0.0128	-0.0104	0.0396	1.14	0.2528
initial BMI	0.3974	0.3262	-0.242	1.0367	1.22	0.2231
Itt: yes	0.2011	1.0675	-1.8912	2.2935	0.19	0.8506
Intercept	-0.0323	0.025	-0.0812	0.0166	1.29	0.1957

Statistics for Model 1

Test of the model: Simultaneous test that all coefficients (excluding intercept) are zero

Q = 2.57, df = 4.0 p = 0.6322
Goodness of fit: Test that unexplained variance is zero
Tau^2 = 0.8879, Tau = 0.9423, I^2 = 94.73%, Q = 56.91, df = 3.0 p = 0.0000

Comparison of Model 1 with the null model

Total between-study variance (intercept only)
Tau^2 = 0.8806, Tau = 0.9384, I^2 = 94.77%, Q = 133.90, df = 7.0 p = 0.0000

Proportion of total between-study variance explained by Model 1

R^2 analog = 0.00 (computed value is -0.01)

Output S4.

Meta-Regression between VLCKD and LDL with Moderators “Proportion of Women in the Study," “BMI at Baseline," “Intention-to-Treat Analysis,” and “Weeks of Intervention"

Main results for Model 1, Random effects (MM), Z-Distribution, Std diff in means

Covariate	Coefficient	Standard	95%	95%	Z-value	2-sided
		Error	Lower	Upper		P-value
		Error	Lower	Upper	P-value	
Intercept	24.5815	18.0448		-10.7858		59.9487
	1.36 0.1731					
women prop	-0.0102	0.0204	-0.0501	0.0297	-0.5	0.6172
initial BMI	-0.7124	0.5231	-1.7378	0.3129	-1.36	0.1732
Itt: yes	1.1489	1.7178	-2.2179	4.5157	0.67	0.5036
Intercept	0.0182	0.04	-0.0601	0.0965	46	0.6482

Statistics for Model 1

Test of the model: Simultaneous test that all coefficients (excluding intercept) are zero

Q = 2.15, df = 4.0 p = 0.7085
Goodness of fit: Test that unexplained variance is zero
Tau² = 2.3754, Tau = 1.5412, I^2 = 97.61%, Q = 125.36, df = 3.0 p = 0.0000

Comparison of Model 1 with the null model

Total between-study variance (intercept only)
Tau² = 1.1359, Tau = 1.0658, I^2 = 95.69%, Q = 162.56, df = 7.0 p = 0.0000

Proportion of total between-study variance explained by Model 1

R^2 analog = 0.00 (computed value is -1.09)

Output S5.

Meta-Regression between VLCKD and TG with Moderators “Proportion of Women in the Study," “BMI at Baseline," “Intention-to-Treat Analysis,” and “Weeks of Intervention"

Main results for Model 1, Random effects (MM), Z-Distribution, Std diff in means

Covariate	Coefficient	Standard	95%	95%	Z-value	2-sided
		Error	Lower	Upper		P-value
		Error	Lower	Upper	P-value	
Intercept	-4.6179	4.6535	-13.7385	4.5028		-0.99
0.321						
women prop	-0.0127	0.0056	-0.0236	-0.0017	-2.27	0.0232
initial BMI	0.1266	0.1343	-0.1366	0.3898	0.94	0.346
Itt: yes	0.6327	0.4286	-0.2074	1.4727	1.48	0.1399
Intercept	-0.0043	0.0103	-0.0245	0.016	-0.41	0.6796

Statistics for Model 1

Test of the model: Simultaneous test that all coefficients (excluding intercept) are zero

Q = 15.38, df = 4.0 p = 0.0040
Goodness of fit: Test that unexplained variance is zero
Tau^2 = 0.0999, Tau = 0.3160, I^2 = 67.23%, Q = 9.15, df = 3.0 p = 0.0273

Comparison of Model 1 with the null model

Total between-study variance (intercept only)
Tau^2 = 0.3721, Tau = 0.6100, I^2 = 88.87%, Q = 62.87, df = 7.0 p = 0.0000

Proportion of total between-study variance explained by Model 1

R^2 analog = 0.73

Figure S1. Funnel Plot – Dependent Variable: BMI

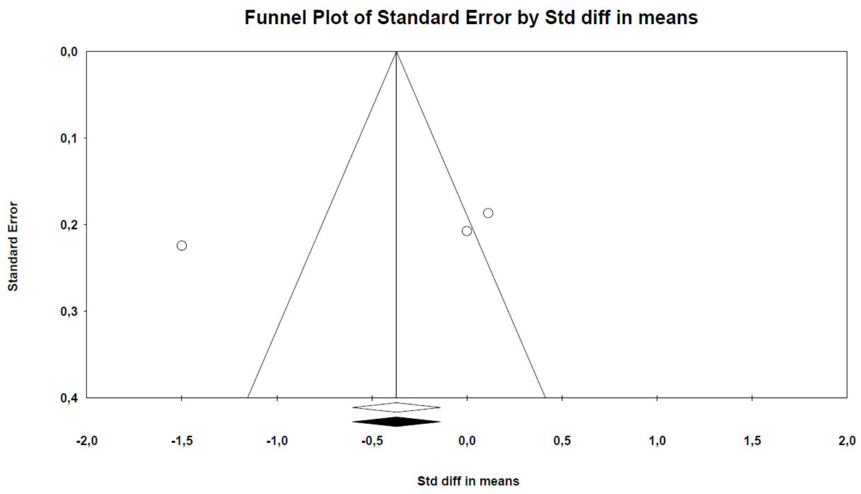

Figure S2. Funnel Plot – Dependent Variable: COL-T

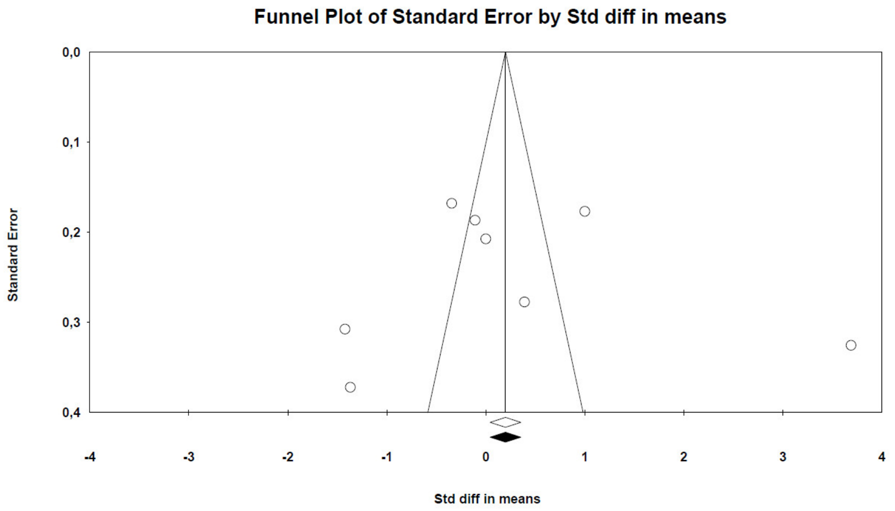

Figure S3. Funnel Plot – Dependent Variable: HDL

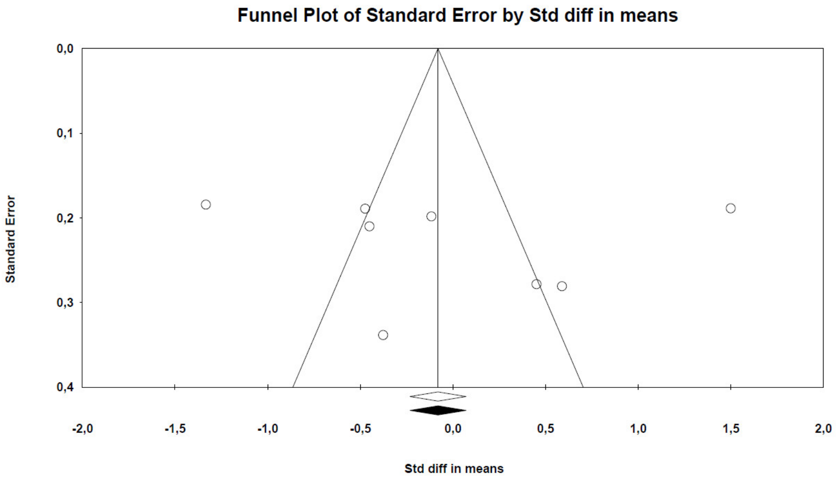

Figure S4. Funnel Plot – Dependent Variable: LDL

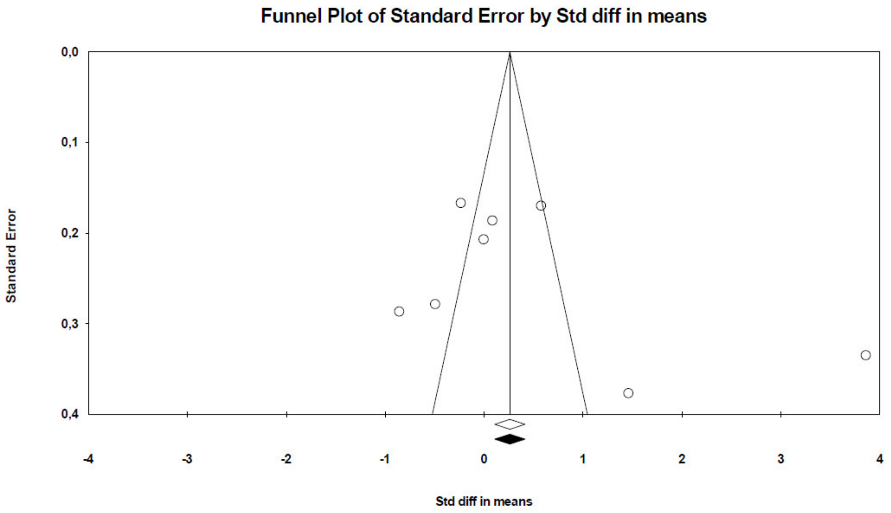

Figure S5. Funnel Plot – Dependent Variable: TG

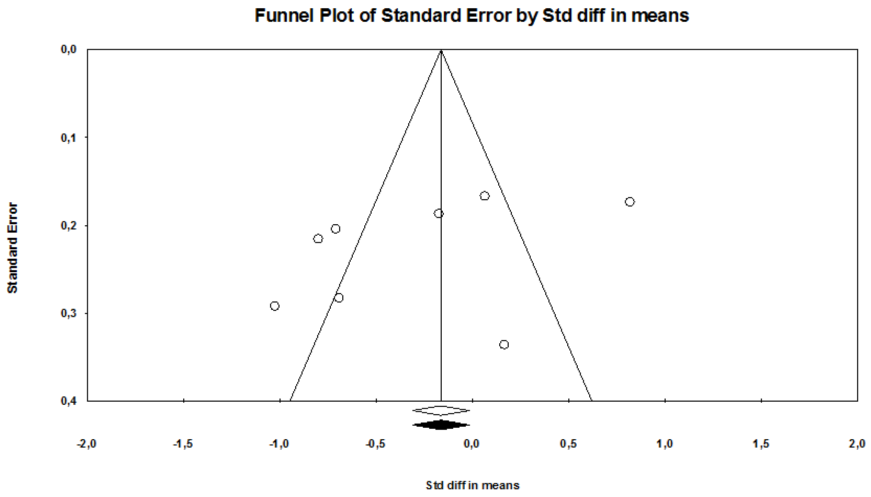

Supplement: Supplementary file 1 [file nutrients-13-02946-s001.zip › nutrients-1338564-supplementary.pdf]
